# COVID-19 and Slums: A Pandemic Highlights Gaps in Knowledge About Urban Poverty

**DOI:** 10.2196/19578

**Published:** 2020-09-04

**Authors:** John Friesen, Peter F Pelz

**Affiliations:** 1 Chair of Fluid Systems Technical University of Darmstadt Darmstadt Germany

**Keywords:** slums, informal settlements, COVID-19, pandemic, infectious disease, living conditions, lifestyle, risk, risk group, health information

## Abstract

According to the United Nations, about 1 billion persons live in so-called slums. Numerous studies have shown that this population is particularly vulnerable to infectious diseases. The current COVID-19 pandemic, caused by the novel coronavirus SARS-CoV-2, emphatically underlines this problem. The often high-density living quarters coupled with a large number of persons per dwelling and the lack of adequate sanitation are reasons why measures to contain the pandemic only work to a limited extent in slums. Furthermore, assignment to risk groups for severe courses of COVID-19 caused by noncommunicable diseases (eg, cardiovascular diseases) is not possible due to inadequate data availability. Information on people living in slums and their health status is either unavailable or only exists for specific regions (eg, Nairobi). We argue that one of the greatest problems with regard to the COVID-19 pandemic in the context of slums in the Global South is the lack of data on the number of people, their living conditions, and their health status.

## Introduction

The spread of SARS-CoV-2 poses one of the greatest challenges to humankind in recent history. According to current estimates (as of July 23, 2020) from Johns Hopkins University [1], there are over 15 million known infections and about 624,000 deaths worldwide in connection with the COVID-19 pandemic.

In the beginning, most COVID-19 infections occurred in countries of the Global North (eg, the United States, Spain, Italy, etc); however, the focus of the pandemic is now shifting toward countries of the Global South (eg, Brazil, India, South Africa, Peru, Chile, and Pakistan). Although only 5% of all confirmed cases (approximately 769,000 as of July 23, 2020) come from African countries, it can be assumed that the number of cases in this region will continue to increase with potentially serious consequences due to limited medical resources [2]. Many African as well as some South Asian countries have the lowest income in the world, with a large proportion of the population living in precarious housing situations often referred to as “slums,” “informal settlements,” or “deprived areas.” According to United Nations estimates, about 1 billion people worldwide currently live in slums [3].

The socioeconomic situation of a person or a group may influence the course of COVID-19. Previous analyses in the United States have shown that socioeconomically disadvantaged groups are at more risk, since they are more frequently affected by the comorbidities that lead to a severe course of COVID-19 compared to the rest of the population [4]. These risk factors are cardiovascular diseases, high blood pressure, diabetes, as well as previous diseases or damage to the lungs [5]. For example, relative to the total population, Black individuals are more likely to experience a severe course of COVID-19 than their White counterparts [6] (the terminology used here—“Black” and “White”—corresponds to that used by Garg et al [6]). Similar distributions of serious cases have been reported in other countries, such as the United Kingdom [7].

Since many studies have shown that slum dwellers are socioeconomically disadvantaged (eg, [8,9]), we need to assess what we know about this group of people in order to determine the risk posed by the virus. We must also examine what we do not know and what we should know.

## What We Know

There is much to be said about the fact that the COVID-19 threat is particularly prevalent in low-income countries, particularly the poorer parts of the population; inhabitants of slums could be especially impacted by the pandemic [10]. There are several reasons for this.

Firstly, social distancing, which is currently being implemented on a large scale across the world, is a physical impossibility in slums due to the high density of buildings and persons per dwelling [11,12]. Furthermore, we know that residents of low- and middle-income countries have increased risks for respiratory infections due to elevated levels of air pollution [13].

As Dahab et al [10] pointed out, if the pandemic enters the slums, their occupants could be much more threatened by severe disease outcomes due to the higher transmissibility of the disease, higher infection-to-case ratios, and higher case fatality. They also demonstrate which measures should be taken, which are realistic (eg, shielding at different levels like households, streets, or blocks), and which are not (eg, tracking of patients) to protect underprivileged and underserved areas [10].

Corburn et al [14] discussed how slums and informal settlements are poorly prepared to manage the pandemic and offered suggestions to minimize the risk of the virus. This is not only a matter of treating the inhabitants of informal settlements on a level equal to the rest of the population but also involves providing them with special support in order to adequately counter the risks associated with their living conditions [15].

## What We Do Not Know

In addition to the studies mentioned above, the pandemic highlights very clearly how limited our knowledge of the living conditions of slums is. We outline below three areas where information is lacking.

1. *Distribution of risk factors among slum dwellers.* Different studies show that the above-mentioned risk factors for severe diseases are not well researched. Data are especially limited on noncommunicable diseases that lead to severe courses of COVID-19 [16]. Although there are many studies on infectious diseases, findings on risk groups, such as people with cardiovascular diseases, are scarce and sometimes contradictory [17,18]. Therefore, we do not know how dangerous the virus is, particularly for these groups.
2. *Regional similarities and differences.* Recent, largescale reviews showed that our knowledge of the health of slum inhabitants is very limited [16,19]. What we know is mostly limited to individual regions, such as Nairobi and Kenya [20]. These findings are confirmed when researching current measures to determine the extent to which countries are taking measures to protect their vulnerable populations. In Nairobi, for example, special attention is given to inhabitants of informal settlements and attempts are made to respond to their needs in the best possible way [21]. Looking at the current data situation, however, Nairobi seems to be an exception. We do not know whether health authorities in other countries and cities have such data or whether they are inaccessible to outside researchers.
3. *Number of inhabitants.* Estimates of residents living in these settlements often differ substantially. Taubenböck et al [22] reported that population estimates for Mumbai vary by a factor of 5 (ie, between 200,000 and 1,000,000 people). Without information on inhabitants, no adequate measures can be taken, both in terms of patient follow-up and in terms of providing necessary care for patients with severe courses of disease. Unreliable population estimates means we are unable to assess the capacity required to deliver these services.

The pandemic unequivocally underlines that we know too little about this vulnerable part of the world’s population, their living conditions, state of health, and thus their inclusion in COVID-19 risk groups. In order to initiate appropriate countermeasures to contain the pandemic, adequate information is necessary.

## What We Should Know

As mentioned above, researchers have already developed proposals to prevent or contain the spread of COVID-19 in slums in a very concrete way [10,14]. Beyond this, however, we think that the following long-term measures are necessary:

1. *Analyses of slum populations and their surroundings.* Recent publications have repeatedly pointed out that it is necessary to identify and classify both individual households and larger low-resource urban areas using uniform frameworks [23]. For this purpose, in addition to the large number of studies that have been conducted in different locations, it is necessary to establish common databases to collect information on the population and spatial characteristics of these settlements.
2. * Detailed comparative research on slum dwellers’ state of health.* It is necessary to investigate what commonalities and differences in the health status of slum dwellers exist [20]. This refers both to the differences within a city between groups living in formal and informal settlements [24], which was done for HIV in South Africa [25], and to the differences across slums in different global regions. The aim here is to become aware of cultural, economic, geographical, infrastructural, religious, or other circumstances, their influences on the health of occupants, and the associated allocation to risk groups. The local population should be involved in information gathering, which can be supported by modern mobile health concepts [26,27]. It is necessary that regionally appropriate countermeasures be taken in the event of challenges such as the current pandemic.

Although these measures are of a longer-term nature and cannot be achieved during the pandemic, they do indicate a way of making visible again the part of the world population that is currently invisible.

## References

[1] Dong E, Du H, Gardner L (2020). An interactive web-based dashboard to track COVID-19 in real time. The Lancet Infectious Diseases.

[2] Gilbert M, Pullano G, Pinotti F, Valdano E, Poletto C, Boëlle P, D'Ortenzio E, Yazdanpanah Y, Eholie SP, Altmann M, Gutierrez B, Kraemer MUG, Colizza V (2020). Preparedness and vulnerability of African countries against importations of COVID-19: a modelling study. The Lancet.

[3] (2016). Urbanization and development: emerging futures. World cities report. United Nations.

[4] Howard G, Safford MM, Moy CS, Howard VJ, Kleindorfer DO, Unverzagt FW, Soliman EZ, Flaherty ML, McClure LA, Lackland DT, Wadley VG, Pulley L, Cushman M (2017). Racial Differences in the Incidence of Cardiovascular Risk Factors in Older Black and White Adults. J Am Geriatr Soc.

[5] Jordan RE, Adab P, Cheng KK (2020). Covid-19: risk factors for severe disease and death. BMJ.

[6] Garg S, Kim L, Whitaker M, O'Halloran Alissa, Cummings C, Holstein R, Prill M, Chai SJ, Kirley PD, Alden NB, Kawasaki B, Yousey-Hindes K, Niccolai L, Anderson EJ, Openo KP, Weigel A, Monroe ML, Ryan P, Henderson J, Kim S, Como-Sabetti K, Lynfield R, Sosin D, Torres S, Muse A, Bennett NM, Billing L, Sutton M, West N, Schaffner W, Talbot HK, Aquino C, George A, Budd A, Brammer L, Langley G, Hall AJ, Fry A (2020). Hospitalization Rates and Characteristics of Patients Hospitalized with Laboratory-Confirmed Coronavirus Disease 2019 - COVID-NET, 14 States, March 1-30, 2020. MMWR Morb Mortal Wkly Rep.

[7] Bhala N, Curry G, Martineau AR, Agyemang C, Bhopal R (2020). Sharpening the global focus on ethnicity and race in the time of COVID-19. The Lancet.

[8] Davis M (2006). Planet of slums.

[9] Wurm M, Taubenböck H (2017). Detecting social groups from space – Assessment of remote sensing-based mapped morphological slums using income data. Remote Sensing Letters.

[10] Dahab M, van Zandvoort K, Flasche S, Warsame A, Ratnayake R, Favas C, Spiegel PB, Waldman RJ, Checchi F (2020). COVID-19 control in low-income settings and displaced populations: what can realistically be done?. Confl Health.

[11] Gibson Lesley, Rush David (2020). Novel Coronavirus in Cape Town Informal Settlements: Feasibility of Using Informal Dwelling Outlines to Identify High Risk Areas for COVID-19 Transmission From A Social Distancing Perspective. JMIR Public Health Surveill.

[12] Wasdani KP, Prasad A (2020). The impossibility of social distancing among the urban poor: the case of an Indian slum in the times of COVID-19. Local Environment.

[13] Gordon SB, Bruce NG, Grigg J, Hibberd PL, Kurmi OP, Lam KH, Mortimer K, Asante KP, Balakrishnan K, Balmes J, Bar-Zeev N, Bates MN, Breysse PN, Buist S, Chen Z, Havens D, Jack D, Jindal S, Kan H, Mehta S, Moschovis P, Naeher L, Patel A, Perez-Padilla R, Pope D, Rylance J, Semple S, Martin WJ (2014). Respiratory risks from household air pollution in low and middle income countries. The Lancet Respiratory Medicine.

[14] Corburn J, Vlahov D, Mberu B, Riley L, Caiaffa WT, Rashid SF, Ko A, Patel S, Jukur S, Martínez-Herrera Eliana, Jayasinghe S, Agarwal S, Nguendo-Yongsi B, Weru J, Ouma S, Edmundo K, Oni T, Ayad H (2020). Slum Health: Arresting COVID-19 and Improving Well-Being in Urban Informal Settlements. J Urban Health.

[15] Buckley RM (2020). Targeting the World's Slums as Fat Tails in the Distribution of COVID-19 Cases. J Urban Health.

[16] Ezeh A, Oyebode O, Satterthwaite D, Chen Y, Ndugwa R, Sartori J, Mberu B, Melendez-Torres GJ, Haregu T, Watson SI, Caiaffa W, Capon A, Lilford RJ (2017). The history, geography, and sociology of slums and the health problems of people who live in slums. The Lancet.

[17] Joshi MD, Ayah R, Njau EK, Wanjiru R, Kayima JK, Njeru EK, Mutai KK (2014). Prevalence of hypertension and associated cardiovascular risk factors in an urban slum in Nairobi, Kenya: a population-based survey. BMC Public Health.

[18] Vusirikala A, Wekesah F, Kyobutungi C, Oyebode O (2019). Assessment of cardiovascular risk in a slum population in Kenya: use of World Health Organisation/International Society of Hypertension (WHO/ISH) risk prediction charts - secondary analyses of a household survey. BMJ Open.

[19] Lilford R, Kyobutungi C, Ndugwa R, Sartori J, Watson SI, Sliuzas R, Kuffer M, Hofer T, Porto de Albuquerque J, Ezeh A (2019). Because space matters: conceptual framework to help distinguish slum from non-slum urban areas. BMJ Glob Health.

[20] Friesen J, Friesen V, Dietrich I, Pelz PF (2020). Slums, Space, and State of Health-A Link between Settlement Morphology and Health Data. Int J Environ Res Public Health.

[21] Abuya T, Austrian K, Isaac A, Kangwana B, Mbushi F, Muluve E, Mwanga D (2020). COVID-19-related knowledge, attitudes, and practices in urban slums in Nairobi, Kenya: Study description. Poverty, Gender, and Youth.

[22] Taubenböck H, Wurm M, Taubenböck H, Wurm M, Esch T, Dech S (2015). Ich weiß, dass ich nichts weiß – Bevölkerungsschätzung in der Megacity Mumbai. Globale Urbanisierung.

[23] Thomson DR, Kuffer M, Boo G, Hati B, Grippa T, Elsey H, Linard C, Mahabir R, Kyobutungi C, Maviti J, Mwaniki D, Ndugwa R, Makau J, Sliuzas R, Cheruiyot S, Nyambuga K, Mboga N, Kimani NW, de Albuquerque JP, Kabaria C (2020). Need for an Integrated Deprived Area “Slum” Mapping System (IDEAMAPS) in Low- and Middle-Income Countries (LMICs). Social Sciences.

[24] Mberu BU, Haregu TN, Kyobutungi C, Ezeh AC (2016). Health and health-related indicators in slum, rural, and urban communities: a comparative analysis. Glob Health Action.

[25] Gibbs A, Reddy T, Dunkle K, Jewkes R (2020). HIV-Prevalence in South Africa by settlement type: A repeat population-based cross-sectional analysis of men and women. PLoS One.

[26] Mechael P, Kaonga NN, Chandrasekharan S, Prakash MP, Peter J, Ganju A, Murthy N (2019). The Elusive Path Toward Measuring Health Outcomes: Lessons Learned From a Pseudo-Randomized Controlled Trial of a Large-Scale Mobile Health Initiative. JMIR Mhealth Uhealth.

[27] Joshi A, Malhotra B, Amadi C, Loomba M, Misra A, Sharma S, Arora A, Amatya J (2020). Gender and the Digital Divide Across Urban Slums of New Delhi, India: Cross-Sectional Study. J Med Internet Res.

